# Merkel Cell Polyomavirus in Respiratory Tract Secretions

**DOI:** 10.3201/eid1503.081206

**Published:** 2009-03

**Authors:** Shan Goh, Cecilia Lindau, Annika Tiveljung-Lindell, Tobias Allander

**Affiliations:** Karolinska Institutet, Stockholm, Sweden; Karolinska University Hospital, Stockholm

**Keywords:** Merkel cell polyomavirus, nasopharyngeal aspirate, real-time PCR, age-related prevalence, dispatch

## Abstract

Merkel cell polyomavirus (MCPyV), associated with Merkel cell carcinoma, was detected in 27 of 635 nasopharyngeal aspirate samples by real-time PCR. MCPyV was more commonly found in adults than in children. Presence in the upper respiratory tract may be a general property of human PyVs.

Polyomaviruses (PyVs) are highly prevalent, small DNA viruses, capable of persistence in the host. To date, 5 human PyVs have been described: JC (JCPyV), BK (BKPyV), KI (KIPyV), WU (WUPyV), and Merkel cell (MCPyV). The discovery of KIPyV and WUPyV in respiratory tract samples has led to many studies of the role of these viruses in respiratory tract disease (*1*–*4*). Moreover, JCPyV and BKPyV viral DNA has been detected in tonsils (*5*,*6*), and BKPyV has been found in respiratory tract secretions (*7*). MCPyV was reported in 2008 and was identified in Merkel cell tumors, a rare form of skin cancer (*8*). We hypothesized that presence in the upper respiratory tract is a trait shared by all human PyVs and investigated whether MCPyV could also be found in respiratory secretions.

## The Study

We used 635 of 637 NPA extracts that had been collected and stored as part of a previous study. Two extracts were insufficient for analysis. A total of 340 samples were from children (median age 5 months, range 10 days–3 years), and 295 samples were from adults (median age 59 years, range 16–93 years). The samples had been sent to Karolinska University Hospital for diagnosis of respiratory tract infections in 2004–2005. Patient identifiers were removed, and the only available clinical information was the patient’s age and sex, month of sampling, and name of referring clinic.

An initial screening by nested PCR with the published MCPyV primer sets LT3, LT1, and VP1 (*8*) identified a strongly positive sample, NPA370, that was used as a positive control for subsequent experiments. Two hydrolysis probe–based, real-time PCRs (rtPCRs) were designed to target the large T antigen (LT) gene and the capsid VP1 gene of MCPyV. Primers and probe targeting the LT gene were (LT.1F) 5′-ccacagccagagctcttcct-3′, (LT.1R) 5′-tggtggtctcctctctgctactg-3′, and (LT probe) 5′-FAM-tccttctcagcgtcccaggcttca-TAMRA-3′. The resulting amplicon was 146 bp. Primers and probe targeting the VP1 gene were (VP1.1F) 5′-tgcctcccacatctgcaat-3′, (VP1.1R) 5′-gtgtctctgccaatgctaaatga-3′, and (VP1 probe) 5′-6FAM-tgtcacaggtaatatc-MGBNFQ-3′. The resulting amplicon was 59 bp. Reactions were performed in 20 μL of 1×TaqMan Universal PCR Master Mix (Applied Biosystems, Foster City, CA, USA), 900 nmol/L of LT.1 primers or 450 nmol/L of VP1.1 primers, 250 nmol/L of LT probe or 500 nmol/L of VP1 probe, and 5 μL of template. Cycling conditions were 50°C for 2 min, 95°C for 10 min, 45 cycles at 95°C for 5 s, and 60°C (LT assay) or 58°C (VP1 assay) for 1 min in a Roche Lightcycler 480 (Roche, Basel, Switzerland).

Due to a limited amount of the positive sample NPA370, control plasmids were constructed for both assays by cloning amplicons of NPA370 into pCR4-TOPO (Invitrogen, Carlsbad, CA, USA): pMCPyVLT.1 containing a 258-bp LT gene amplicon (FJ472933) and pMCPyVVP1.1 containing a 179-bp VP1 gene amplicon (FJ472932). Serial dilutions of the plasmids were used to determine assay sensitivity, and pMCPyVLT.1 was also used to determine a genome copy number correlate for the LT assay. In both assays, plasmid control with 2 copies/reaction was reproducibly positive, corresponding to 400 copies/mL of sample. Specificity of both assays was assessed by a range of templates: a plasmid containing the complete KIPyV genome; a WUPyV-positive sample NPA213; 4 urine samples positive for either BKPyV or JCPyV; and a panel of samples containing respiratory syncytial virus, influenza A and B viruses, adenovirus, bocavirus, parainfluenza virus, metapneumovirus, herpes simplex virus type 1 and 2, varicella-zoster virus, human herpesvirus 6, parvovirus B19, cytomegalovirus, echovirus 30, *Mycoplasma pneumonie*, *Chlamydophila pneumoniae*, and *Legionella pneumophila*. All the above samples were negative by LT and VP1 assays. To check for contamination, we included at least 4 water controls per run of 10–86 samples; no amplification was observed.

Of the 635 NPA samples, 44 (6.9%) were positive for MCPyV DNA by the LT assay, 84 (13.2%) were positive by the VP1 assay, and 27 (4.3%) were positive by both assays. With a few exceptions, viral DNA copy numbers were low, as determined by cycle threshold values (mean LT/VP1 = 38.6/39.0) and plasmid equivalent counts of the LT assay (Table). To further validate these findings, 10 double-positive samples and all LT-positive (+)/VP1-negative (–) samples were amplified by conventional PCR using the LT primers. All double-positive samples gave the expected 146-bp product, which was confirmed to have MCPyV sequence (FJ472034–43), but the LT+/VP1– samples did not generate specific products. Whether this was due to lower sensitivity of the conventional PCR or occasional unspecific amplification in the LT rtPCR could not be determined. The VP1 PCR product was too short to enable direct sequencing. Therefore, only samples positive by both assays were considered positive for MCPyV.

**Table:** Consensus results of 2 real-time PCRs for MCPyV in adults and children*

pMCPyVLT.1 equivalents		No. samples	No. children (<15 y)	No. adults (>15 y)	No. male	No. female	Coinfection†
Per reaction	Per mL of sample						
<2 (negative)	<400 (negative)	608	338	269	308	299	229‡
2–100	400–20,000	22	2	20	15	8	7§
101–617	20,200–123,000	5¶	0	5	4	1	1#

*MCPyV, Merkel cell polyomavirus. †Viruses detected by immunofluorescence, virus culture, and real-time PCR. ‡Adenovirus, enterovirus, herpes simplex virus type 1, influenza A virus, influenza B virus, KI polyomavirus, metapneumovirus, parainfluenza virus type 1 and 3, respiratory syncytial virus, WU polyomavirus. §Influenza A and B viruses. ¶Includes NPA370. #Influenza virus A.

By these criteria, 25 of 295 samples from adults and 2 of 340 samples from children were positive. Therefore, significantly more adults than children were MCPyV positive (p<0.001, χ^2^ test). MCPyV was particularly prevalent among the elderly. The median age for MCPyV carriers was 74 years. A skew in gender in MCPyV carriers was not statistically significant. None of the MCPyV-positive samples were positive for KIPyV or WUPyV.

## Conclusions

MCPyV DNA was detected in 4.3% of NPA samples from patients with respiratory disease as determined by combined results of 2 rtPCRs. Viral DNA was detected in low copy numbers, which makes reproducibility of positive results challenging (*9*). Therefore, a prevalence rate of 4.3% is probably an underestimation of samples containing MCPyV DNA at concentrations below the reproducible detection limit. A similar result of discordant LT and VP1 positive Merkel cell tumor samples has been reported (*8*). Contamination by the plasmid template is unlikely because water controls were consistently negative and LT and VP1 positivity were significantly associated (p<0.001, χ^2^ test). Notably, both assays were easily reproducible for the 5 samples that had relatively high copy numbers (Table).

The prevalence of MCPyV in this collection of NPA samples is higher than that of KIPyV and WUPyV (*10*). Also, significantly more adults (particularly the elderly) were MCPyV positive than were children. This result is in contrast to results with KIPyV and WUPyV, which have mainly been found in children (*1*,*2*). Possible reasons for this age-related prevalence include incidence increases with exposure time, activation of latent virus increases with age, and possible previous exposure to transmission of MCPyV that is no longer in effect (*11*). Alternatively, a larger number of adult patients may be immunosuppressed than are the children. However, most MCPyV-positive samples (22/27) were sent for influenza diagnostics from the infectious diseases unit, not a hematology or oncology unit; hence, patients were probably not severely immunosuppressed.

The presence of MCPyV in respiratory secretions indicates that it is shed into the respiratory tract or present in cells of the respiratory tract, similar to KIPyV, WUPyV, BKPyV, and possibly JCPyV. Its presence also suggests that the respiratory tract may be a route of transmission. The use of stored nucleic acid extracts did not enable us to determine whether detected MCPyV DNA was intracellular or virion-associated. Thus, the newly discovered human PyVs can all be found in the respiratory tract. Conclusions about their primary target organs and pathogenicity cannot be drawn without epidemiologic support and further investigation on different sample types.
